# Selection and validation of reference genes for quantitative gene expression analyses in black locust (*Robinia pseudoacacia* L.) using real-time quantitative PCR

**DOI:** 10.1371/journal.pone.0193076

**Published:** 2018-03-12

**Authors:** Jinxing Wang, Manzar Abbas, Yanzhong Wen, Dongsheng Niu, Ling Wang, Yuhan Sun, Yun Li

**Affiliations:** 1 Beijing Advanced Innovation Center for Tree Breeding by Molecular Design, National Engineering Laboratory for Tree Breeding, College of Biological Sciences and Technology, Beijing Forestry University, Beijing, People's Republic of China; 2 Propagation and Breeding Farm for Improved Forest Trees of Jixian County, Shanxi Province, Linfen, People's Republic of China; 3 Badaling Forestry Farm of Beijing, Beijing, People's Republic of China; USDA Agricultural Research Service, UNITED STATES

## Abstract

Black locust (*Robinia pseudoacacia* L.) is an easy to raise, fast growing, medium-sized deciduous tree species highly tolerant to harsh eco-conditions, i.e., drought and harsh winters, and it is widely adaptable to sandy, loamy, and marshy soils. The basis for this adaptability remains to be investigated at the transcriptomic level using real-time quantitative PCR (qPCR). Selection of a reliable gene for the normalization of qPCR data is important for obtaining accurate results in gene expression. The goal of this study was to identify an appropriate reference gene from 12 candidate genes for gene expression analysis in black locust exposed to various stressors such as abscisic acid (ABA), NaCl, polyethylene glycol (PEG) and varying temperatures. In GeNorm and NormFinder analyses, *ACT* (actin) and *GAPDH* (glyceraldehyde-3-phosphate dehydrogenase) gene expression were the most stable in all conditions except heat stress, but in BestKeeper analysis, *GAPDH* and *helicase* gene expression were the most stable under NaCl and heat stress. In contrast, *ACT* and *GAPDH* were highest under abscisic acid (ABA), *GAPDH* and *βTUB* (beta tubulin) under cold stress, and *helicase* and *EF1α* (elongation factor 1 alpha) under PEG stress. We found that the most stable reference gene combination for all conditions was *ACT* and *GAPDH*. Additionally, the expression pattern of *NAC2* (a transcription factor) and *BGL2* in different tissues and under different stress conditions was analyzed relative to *ACT* and *GAPDH* and *UBQ* (ubiquitin) the least stably expressed gene. *NAC2 and BGL2* both had highest expression in flowers and pods under ABA stress at 48h. This study provides useful reference genes for future gene expression studies in black locust.

## Introduction

Black locust (*Robinia pseudoacacia* L.) is a monoclinous leguminous tree belonging to the Faboideae subfamily. It is widely cultivated in the southeastern United States, temperate Europe, and Asia [1] and is of ecological and economic value [2,3]. It is also used as forage, wood fiber, feed stock, lumber, fuel and in beekeeping [3,4,5]. Black locust was first introduced to China in 1877‒1878, and currently is extensively cultivated in many parts of the country [6]. In addition, black locust flowers can be used by honeybees to produce honey and royal jelly [5]. In the semi-arid Loess Plateau of China, it is also extensively being used for reforestation [7]. Consequently, black locust is an ecologically and economically valuable species.

Many biological techniques have been used to confirm the expression levels of genes, such as RNase protection assays (RPAs), Northern blotting, real-time quantitative PCR (qPCR), gene chips (DNA microarray), fluorescent in situ hybridization (FISH), serial analysis of gene expression (SAGE), tilling arrays, RNA sequencing, reporter genes, digital droplet PCR (ddPCR) and semi-quantitative PCR (semi-PCR). RNA sequencing and qPCR are considered the best methods due to their high throughput nature [8,9]. Northern blotting was the first RNA-based technique to allow measurement of differential gene expression. Northern blotting is time consuming and laborious, and most importantly, accurate quantification of expression levels is poor using this method. Another important gene expression technique is DNA microarray hybridization, which uses either cDNA or oligonucleotides. Microarray analysis has the ability to screen a large number of target genes, even under multiple stimuli [10]. Microarrays utilize plates spotted with DNA sequences representing each target gene. In this process, template cDNA from control and fluorophore-labeled samples are hybridized to the glass plate. The difference between fluorescent signal intensities represents differential gene expression; however, poor or weak signals generation can complicate gene expression analysis of low expression genes. Reporter genes such as *lacZ*, *cat*, luciferase, *GUS*, *GFP* and *rfp* can also be considered but they are time consuming and laborious, because they require genetic manipulation and successful transformation events.

The leading gene expression techniques for achieving authentic and reproducible results are RNA sequencing and qPCR [11]. They provides better results compared with other RNA quantification techniques because of its accurate quantification and high sensitivity [8,11,12]. As a result, the interpretation of differential gene expression patterns can lead to a better understanding of biological processes. Consequently, given the accuracy, high sensitivity, and reproducibility qPCR has become the standard method for in-depth studies of gene expression, alternative splicing events, validation of microarray data, and medical diagnostics [13–21]. Different non-specific factors present challenges for normalization of gene expression including differences in the nature and quantity of RNA, some of which can be controlled by establishing candidate reference genes [22,23].

In the past few years, ubiquitin (*UBQ*), elongation factor 1 alpha (*EF1α*) [24], and glyceraldehyde-3-phosphate dehydrogenase (*GAPDH*) [25], have been used frequently as internal controls for qPCR studies. Many studies revealed that these genes exhibit non-uniform and non-stable expression, highlighting the demand for better universal internal control genes [26,27]. With this goal in mind, a number of qPCR control genes have been reported in diverse plant species including tobacco [28], Arabidopsis [29,30], berry [31], poplar [32–34],eucalyptus [35,36], and teak [37]. To determine which reference gene is best for transcript normalization in a given subset of biological samples, statistical algorithms such as RefFinder, NormqPCR, RefGenes, OLIVER, geNorm, BestKeeper, and NormFinder have been developed (https://omictools.com/reference-genes-category) [38,39].

We used statistical and graphical methods to compare the expression stability of 12 candidate reference genes (Table 1) across a large set of organs i.e., root, stem, leaf, flower, pod and stress treatments (ABA, NaCl, PEG and variable temperature conditions). The expression stability of these genes was subsequently evaluated using geNorm [40], BestKeeper [38], and NormFinder [41]. Furthermore, the expression patterns of the target genes were investigated using the selected reference genes along with transcription factor *NAC2* which is involved in protecting plants against abiotic stresses, flowering, senescence [41,42] and *BGL2*, which is also involved in flowering. This study provides the most extensive analysis of potential reference genes that can be applied to future gene expression studies in black locust.

**Table 1:** Descriptions of the 12 candidate reference genes and two selected genes for validation of results.

Symbol	Gene Name	GeneBank Accession Number	Arabidopsis homolog locus	Function
*SAMDC*	*S-adenosylmethionine decarboxylase*	KJ587744	*AT3G02470*	Involved in polyamine biosynthesis.
*18S rRNA*	*18S ribosomal RNA*	KJ587743	*AT2G47990*	Constituent of the small ribosomal subunit.
*ACT*	*Actin*	KJ587742	*AT1G01750*	Cytoskeletal structural protein.
*EF1α*	*Elongation factor 1 alpha*	KJ587747	*AT1G30230*	Protein synthesis.
*GAPDH*	*Glyceraldehyde-3-phosphate dehydrogenase*	KJ587745	*AT1G13440*	Oxidoreductase in glycolysis and gluconeogenesis.
*Helicase*	*Helicase*	KJ587746	*AT2G30800*	RNA processing.
*UBQ*	*Ubiquitin*	MG515604	*AT1G04860*	Protein binding and modification.
*βTUB*	*Beta tubulin*	MG515605	*AT1G04820*	Major constituent of microtubules.
*CYP*	*Cyclophilin*	MG515606	*AT4G34870*	Protein folding.
*SAND*	*Sand family protein*	MG515607	*AT2G*28390	Plants organs development and protein transport.
*CAC*	*Clathrin adaptor complex*	MG515608	*AT5G46630*	Endocytosis, intracellular protein transport.
*PP2A*	*Phosphatase 2A*	MG515609	*AT1G69960*	Regulation of auxin polar transport.
**Target Genes**				
*NAC*	*NAC domain containing protein 2*	MG515610	*AT3G15510*	Flowering, development and stress response.
*BGL*	*beta-1*,*3-glucanase 2*	MG515611	*AT3G57260*	Involved in flowering.

Database source: NCBI Reference Sequence database Genebank batch upload accession number is PRJNA260115 (http://www.ncbi.nlm.nih.gov).

## Materials and methods

### Plant materials and treatments

From June to September 2016, we collected roots, leaves, flowers, pods and stems from 5 year old black locust grown in the Mijiabao Forest Farm, Yanqing District of Beijing, PR of China. Note, no specific permission or grant was required from authorities to collect samples for research purpose as this forest is open for research purposes. Seeds were grown in pots filled with soil mixture consisting of silt, perlite and Floraguard Substrat^®^ (3:1:5) and when the seedlings were two months old, they were pulled carefully and dipped in NaCl (200 mM), PEG-6000 (10%), or ABA (150 μM) for 12, 24 and 48h [43]. For the cold and heat treatments, plants were exposed to 4°C or 40°C by placing whole plants in chambers under a 14-h light/10-h dark photoperiod for 0, 12, 24, and 48 h. A total of 21 samples in triplicate were collected from different organs (roots, leaves, pods, and stems) at varying time points (0, 12, 24, and 48 h) and then immediately chilled in liquid nitrogen and stored at −80°C.

### Total RNA extraction quality control and cDNA synthesis

For total RNA extraction, 2g samples from each tissue were collected, and immediately chilled in liquid nitrogen and grounded in a pre-chilled, autoclaved pestle and mortar. The RNeasy^®^ Plant Mini Kit (Qiagen, Hilden, Germany) was used for extraction of RNA from all samples. Total RNA was treated with RNase free DNase I (Promega, USA) to remove any contaminating DNA. The optical density (OD) of total RNA was determined on a Nanodrop ND-1000 spectrophotometer (Thermo Scientific, Waltham, MA, USA) at an absorbance ratio of 260/280 nm, with RNA samples ranging 1.9 to 2.2. For further analyses, an OD of 2.0 at an absorbance ratio of 260/230 nm was used [44]. RNA integrity was validated by separation of the RNA on a 1.5% (w/v) agarose gel containing ethidium bromide, followed by visualization under UV light in a gel documentation system. RNA samples with an OD of 1.5‒2.0 and clear bands representing 28S/18S rRNA without smearing on the agarose gel were used for cDNA synthesis. The first cDNA strand was synthesized from 1000 ng total RNA in a volume of 20 μl using the PrimeScript^™^ RT reagent Kit with gDNA eraser (Perfect Real Time), (Clontech, Shiga, Japan), according to the manufacturer’s protocol. In the first step, genomic DNA (gDNA) was removed and the integrity of the RNA was verified by running samples on 1.2% agarose gel according to the protocol. In the second step, we used oligo-dT primers to perform reverse transcription. The quantity of cDNA was measured on a ND-1000 Nanodrop spectrophotometer at 260/280nm absorbance and quality was examined by electrophoresis using a 1.2% agarose gel. Newly synthesized cDNA was diluted 10-fold prior to determination of its quality and quantity and stored at -20°C until use for the qPCR study.

### Primer design

Twelve reference genes were selected for primer design: *SAMDC*, *18SrRNA*, *ACT*, *EF1α*, *GAPDH*, *Helicase*, *UBQ*, *βTUB*, *CYP*, *SAND*, *CAC*, and *PP2A* (Table 1). Black locust gene sequences were obtained by transcriptome sequencing in our laboratory. A total of 12 pairs of primers for candidate reference genes, along with two primer pairs for validation of results were designed from CDS sequences (with a maximum amplicon length of 293 bp, optimal Tm of 50‒60°C, GC percentage of 20‒80%, primer length 18‒23bp) using the bioinformatics tool Primer Premier 5.0 (Table 2).

**Table 2:** Primers, amplicon sizes, and qPCR efficiency for the candidate internal control and results validation genes.

*Symbol*	Forward Primer	Reverse Primer	Amplicon Length (bp)	Tm (°C)		qPCR Efficiency
*ACT*	5^′^TTGCCTTGGATTATGAACA3^′^	5^′^ GATGGCTGGAACAGAACTT3^′^	139	84.1		100.2%
*GAPDH*	5^′^TCAACAATGCCAAACCTG3^′^	5^′^GTGTCAACGAGCACGAAT3^′^	115	90.7		101.6%
*18SrRNA*	5^′^ATAAACGATGCCGACCAG3^′^	5^′^G CCTTGCGACCATACTCCC3^′^	105	80.5		99.3%
*EF1α*	5^′^CTGCCAACTTCACATCCC3^′^	5^′^CTTTACCGAACGCCTATC3^′^	146	85		98.5%
*SAMDC*	5^′^CAGCAGAGGCAGAGTAGAC3^′^	5^′^ATTGAAGTTGGCGGAGGG3^′^	221	82.9		97.6%
*Helicase*	5^′^CTGACAAGATGCGAAGCC3^′^	5^′^TCAATACCACGAGCCAAA3^′^	145	83.6		102.1%
*UBQ*	5^′^ACCAACAGCGTCTCATCT3^′^	5^′^GTTATAGTCAGCCAAAGTGC3^′^	293	90.1		100.7%
*bTUB*	5^′^GAGGAATACCCAGACAGA3^′^	5^′^TTTCGGAGGTCAGAGTT3^′^	287	85.7		98.4%
*CYP*	5^′^TCTGTGGTGGCTCTGAAT3^′^	5^′^ACAAGACTCCCACTCTATGA3^′^	185	83.5		101.1%
*SAND*	5^′^CCGATCCAACTCCACTT3^′^	5^′^CCAGATCCGTGAACGAC3^′^	95	87.8		100.8%
*CAC*	5^′^AATCCGCTGACTCGTAAC3^′^	5^′^ TCTCCGACGAAAGGCTAC3^′^	168	88.1		97.5%
*PP2A*	5^′^AGACTCGGGCAGTAAACC3^′^	5^′^TCCCACCTCTGGAGACAC3^′^	168	85.4		98.1%
**Target Gene**						
*NAC2*	5^′^TCAAATAAAGGAAAGAG3^′^	5^′^GGTAGTGAACAACGAGT3^′^	181	84.3		98.1%
*BGL2*	5^′^CATCTCCATTTCCACAC3^′^	5^′^ACAGCAACATCACCATT3^′^	157	83.1		101.7%

Database source: NCBI Reference Sequence database Genebank batch upload accession number is PRJNA260115 (http://www.ncbi.nlm.nih.gov).

### qPCR analysis

Initially all primers were tested by qPCR using Premix Ex Taq^™^ (TaKara) on an ABI 7500 (Applied Biosystems^®^) real-time PCR system. The total volume of the reaction mixture was 20 μl containing 10 μl 2X SYBR Premix Ex Taq^™^, 1 μl of the template cDNA reaction mixture, 0.5 μl of each primer (100 μM), 0.5 μl ROX Reference DyeII, and 7.5 μl double distilled H_2_O (ddH_2_O). The PCR protocol was as follows: pre-denaturation at 95°C for 30 s, denaturation at 95°C for 3 s, annealing and amplification at 58°C for 30 s, 33 cycles in total, and a 55‒95°C melt curve analysis. All reactions were performed in triplicate. The threshold cycles (C_q_) were automatically calculated for each reaction, these values were then used to determine the mean C_q_ for each data set.

### Statistical analysis

For adequate reference gene selection, three Microsoft Excel-based software programs, GeNorm (version 3.5) (http://medgen.ugent.be/*jvdesomp/genorm/), BestKeeper (version 1), and NormFinder (version 0.935), were used to analyze the stability of gene expression. The designer’s instructions and guidelines were followed for all three programs. To apply GeNorm and NormFinder for stable gene expression calculations, C_q_ values were transformed based on the data input format. The lowest C_q_ value, representing the highest expression level was used as control and given a value of 1. For GeNorm and NormFinder analyses, raw data were required to be transformed in standard input format, while for BestKeeper analysis, the only original C_q_ values were used. Standard curves were generated in Microsoft Excel the C_q_ values plotted against the logarithmic quantities of the known amounts of cDNA dilutions: 1, 1/5, 1/25, 1/125, 1/625, and 1/3125 using cDNA adjusted to 200 ng/μl as a start material. To determine the correlation coefficients (r^2^ values), only C_q_ values less than 40 were considered. To determine amplification efficiencies (E) only C_q_ values less than 40 were used in the following formula E = [5^-(1/slope)^-1]×100%. The efficiencies obtained in all qPCR assays were between 95% and 105%. To calculate the relative expression level of each gene, the 2^-△Cq^ method was applied [45]. While, △C_q_ was determined by subtracting the minimum C_q_ value from candidate reference genes C_q_ (△C_q_ = candidate reference gene C_q_ value–minimum C_q_ value). These data was further analyzed by GeNorm and BestKeeper software tools.

Analysis of variance using SPSS (http://xiazai.zol.com.cn/detail/44/437842.shtml) was performed to compare the data at a significance level of P<0.05. To calculate the expression stability values and to identify the most stably expressed reference genes, the Excel-based algorithm BestKeeper was inevitable [28]. For this, the standard deviation (SD), correlation coefficient (r) and coefficient of variance (CV) were estimated. A gene with a SD value >1 was treated as unstable. To identify the highest stably expressed genes, a BestKeeper Index based on the geometric means of the C_q_ values was calculated.

## Results

### Expression profiles of the reference genes

**Fig 1:**
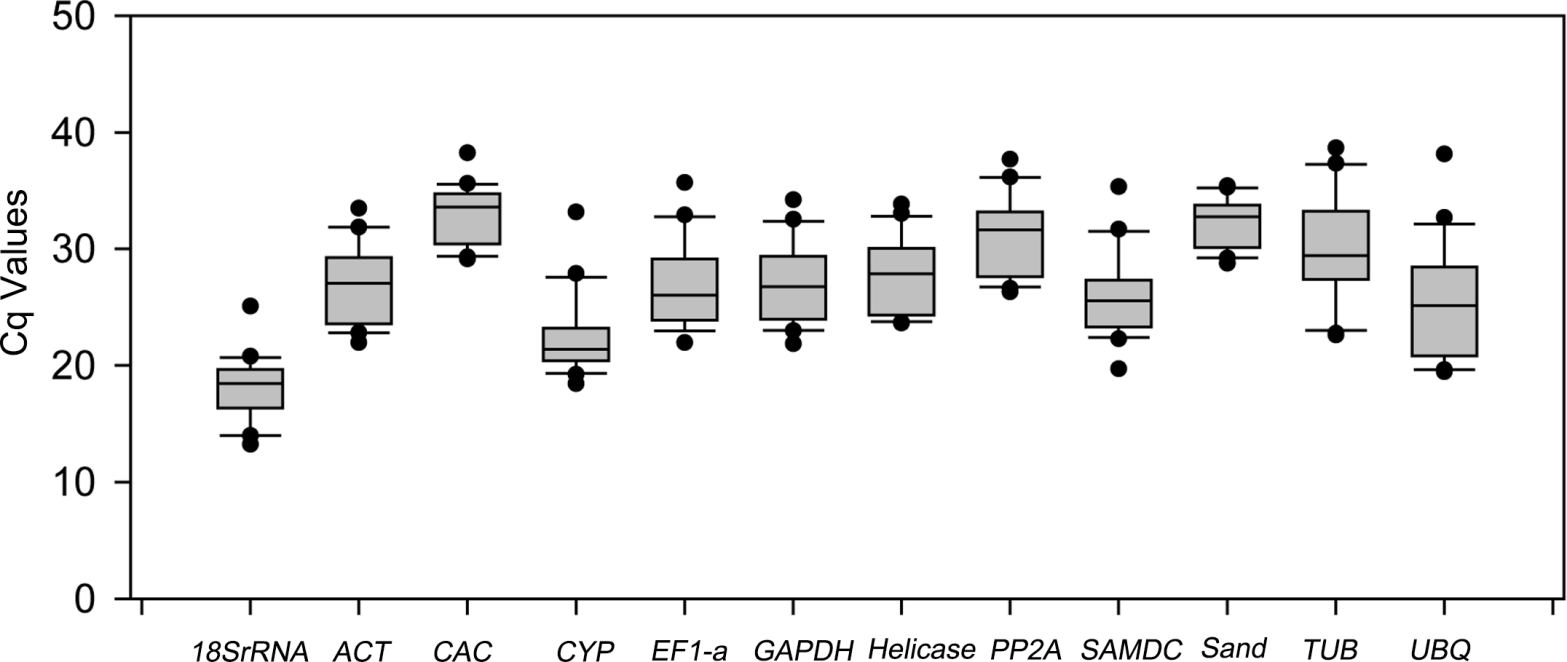
Distribution of the average C_q_ values of all 12 reference genes evaluated in black locust samples: *18SrRNA*, *ACT*, *CAC*, *CYP*, *EF1a*, *GAPDH*, *Helicase*, *PP2A*, *SAMDC*, *SAND*, *βTUB*, and *UBQ*. The linear line shows median, dots for extremes, boxes for 25%-75% C_q_ value and whiskers for 5%-95% respectively.

Of the 12 candidate reference genes, 9 (*ACT*, *GAPDH*, *βTUB*, *UBQ*, *EF1a*, *helicase*, *PP2A*, *18s rRNA*, and *SAMDC*) are traditional control genes, while 3 (*SAND*, *CAC*, and *CYP*) are novel candidates (Table 1). The C_q_ values, representing the cycle at which significant PCR product was amplified, were calculated for each gene (Table 2). Generally, the C_q_ value was obtained in the mid-exponential phase of amplification (Fig 1). The C_q_ values of all 12 potential reference genes varied widely from 13 to 34 cycles. The highest expressed gene in all tissues was *18SrRNA*, with a C_q_ value < 25.

### geNorm analysis

**Fig 2:**
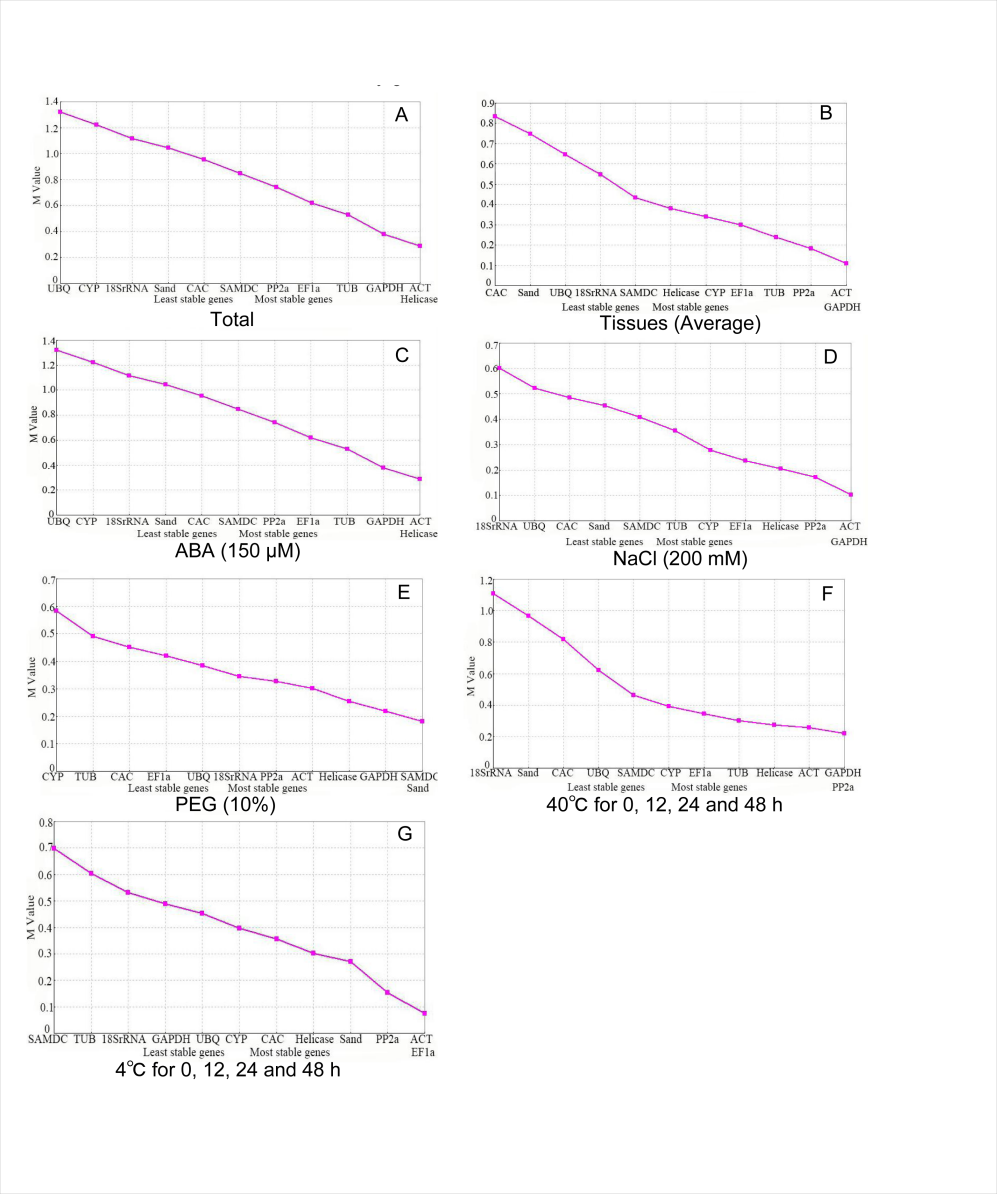
Average expression stability values (M) of 12 candidate reference genes under different conditions and tissues calculated by geNorm.

To analyze and rank the reference genes in terms of expression stability, geNorm was used. C_q_ values were obtained for all samples and transformed into relative quantities using the △ C_q_ method. The stability value M was calculated for the average pairwise expression ratio. A gene with a low M value is considered stable, while a gene with a high M value is considered unstable (Fig 2). To identify potential reference genes, geNorm recommends selecting genes with an M value < 1.5. In combined analyses, *helicase* and *ACT* had the lowest M values (0.037) and thus the highest expression stability, followed by *GAPDH* and *PP2A*, whereas *18s rRNA* and *UBQ* were less stable. When untreated tissues such as root, stem, leaf, flower and pods were analyzed, *ACT* and *EF1a* were most stably expressed, with a minimum M value of 0.01, followed by *SAND* and *helicase*, while *SAMDC* and *TUB* were least stable. Under ABA stress, *ACT* and *GAPDH* were most stable, *TUB* and *EF1a* moderately stable, and *CAC* and *SAND* the least stable. Under NaCl stress, *ACT*, *helicase*, and *GAPDH* were most stable, *TUB* and *EF1a* less stable, and *UBQ* and *CYP* the least stable. *ACT*, *GAPDH*, and *PP2A* were highly stable, *helicase* and *EF1a* were moderately stable, and *18s rRNA* and *UBQ* the least stable under PEG treatment. Under heat stress (40°C), *SAMDC*, *SAND*, and *GAPDH* were relatively stable, *ACT* and *PP2A* less stable, and *CYP* and *TUB* were the least stable. Under cold stress (4°C), *ACT*, *PP2A*, and *GAPDH* were highly stable, *TUB* and *EF1a* were less stable, and *18s rRNA* and *SAND* were the least stable. In conclusion, the most stable reference genes in all samples according to the geNorm analysis were *ACT* and *GAPDH*.

**Fig 3:**
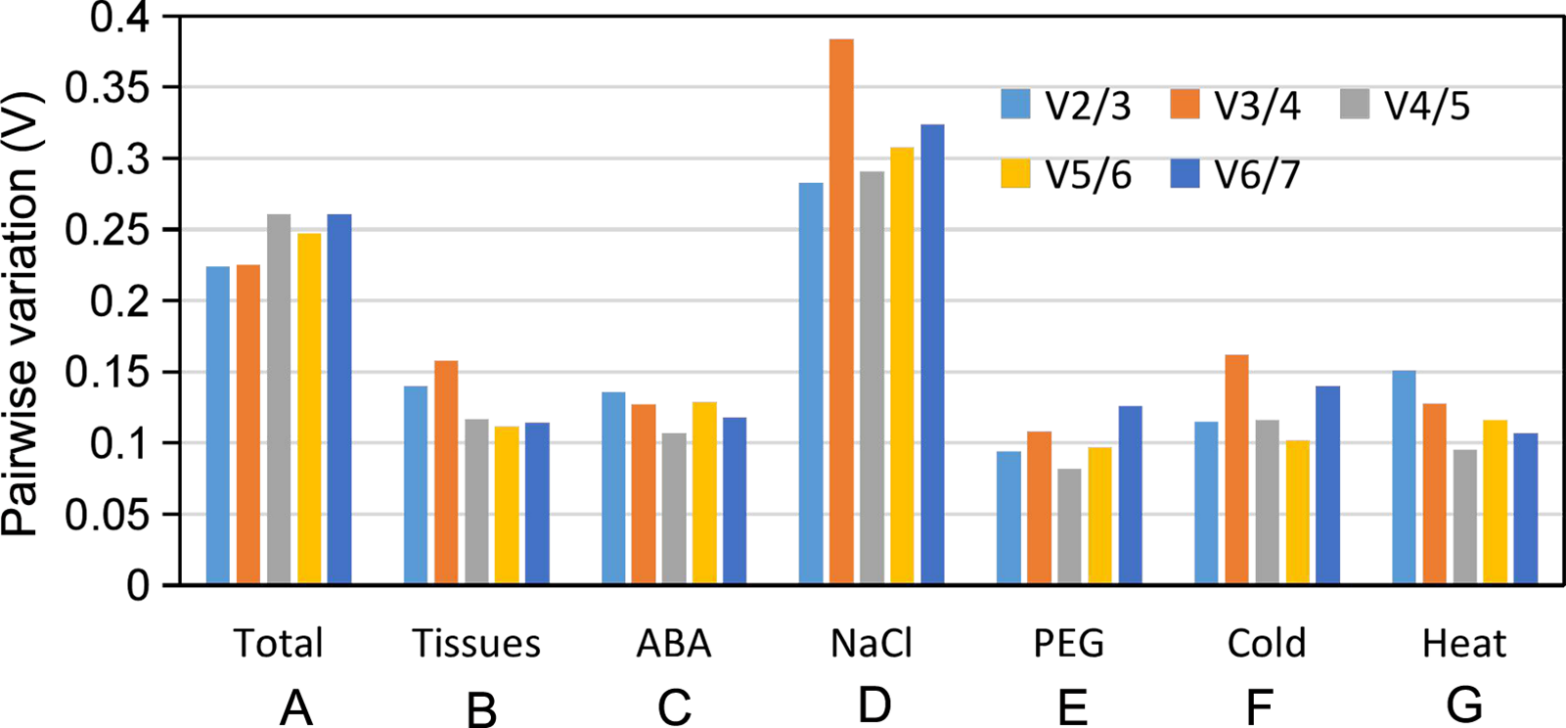
Estimation of the required number of reference genes for normalization by pairwise variation (V) using geNorm.

To minimize interpretation of qPCR expression analyses and to increase overall robustness of the analyses, the use of two internal reference genes is highly recommended [38]. To calculate the number of internal control genes needed, the following formula V_n/n+1<0.15_ (n = 1,2,3…..) was developed [39]. According to the aforementioned formula, if V<0.15, then no inclusion of ≥n+1 reference genes should be considered. The data depicted in Fig 3 indicates that expanding the reference gene set beyond two control genes will not significantly impact the outcome. Surprisingly, NaCl treatment (D) had the greatest variation among all samples.

### NormFinder analysis

NormFinder is another Microsoft Excel-based Visual Basic application that assigns stability values to single candidate reference genes. The NormFinder algorithm uses a model-based approach (M value) for estimation of gene expression variation among candidate genes. NormFinder takes into account intra- and intergroup variation for normalization factor calculations and prevents misinterpretations caused by artificial selection of co-regulated genes.

We used NormFinder to analyze the gene expression profiles of all genes evaluated (Table 3). Although the ranking order of the genes differed somewhat from the geNorm findings, no significant differences were observed. *ACT*, *GAPDH*, and *helicase* were stable candidate reference genes, with M values of 0.16, 0.17, and 0.17, respectively. In plants treated with chemicals ABA, PEG, and NaCl and different temperature conditions (cold and hot), the remaining nine genes (*SAMDC*, *18s rRNA*, *EF1α*, *UBQ*, *βTUB*, *CYP*, *SAND*, *CAC*, and *PP2A*) exhibited large fluctuations in their expression profiles. Those genes that exhibited moderate gene expression in NormFinder exhibited slightly different expression stabilities relative to geNorm. The overall ranking of the candidate reference genes by NormFinder was *ACT* > *GAPDH* > *helicase* > *EF1a* > *PP2A* > *SAMDC* > *βTUB* > *CYP*> *CAC* > *SAND* >*UBQ* > *18s rRNA*. The NormFinder results were extremely stable and consistent compared with the geNorm results.

**Table 3:** Average expression stability values of the 12 reference genes in different organs, calculated by NormFinder.

Rank	Total(A)	Tissues(B)	ABA(C)	PEG(D)	NaCl(E)	Heat(F)	Cold(G)
1 M value	ACT	PP2A	Helicase	PP2A	ACT	GAPDH	Helicase
	0.16	0.09	0.03	0.07	0.13	0.07	0.07
2 M value	GAPDH	ACT	TUB	CYP	GAPDH	SAMDC	PP2A
	0.17	0.11	0.06	0.10	0.14	0.13	0.07
3 M value	Helicase	EF1a	PP2A	Helicase	Helicase	Helicase	ACT
	0.17	0.15	0.12	0.14	0.17	0.14	0.09
4 M value	EF1a	CAC	SAMDC	ACT	SAMDC	ACT	GAPDH
	0.30	0.20	0.18	0.19	0.47	0.19	0.13
5 M value	PP2A	GAPDH	ACT	GAPDH	EF1a	SAND	TUB
	0.37	0.27	0.19	0.22	0.48	0.21	0.28
6 M value	SAMDC	Helicase	GAPDH	SAMDC	TUB	PP2A	EF1a
	0.47	0.29	0.20	0.26	0.58	0.26	0.34
7 M value	TUB	UBQ	CYP	EF1a	18s rRNA	EF1a	CYP
	0.59	0.30	0.38	0.27	0.75	0.27	0.49
8M value	CYP	SAND	EF1a	SAND	CAC	18s rRNA	SAMDC
	0.61	0.33	0.42	0.31	0.78	0.32	0.54
9 M value	CAC	CYP	18s rRNA	TUB	PP2A	TUB	CAC
	0.70	0.34	0.55	0.34	0.81	0.34	0.83
10 M value	SAND	18s rRNA	SAND	CAC	CYP	UBQ	SAND
	0.73	0.49	0.71	0.37	0.98	0.34	0.99
11 M value	UBQ	TUB	UBQ	UBQ	SAND	CAC	UBQ
	0.77	0.57	0.83	0.49	1.02	0.39	1.09
12 M value	18s rRNA	SAMDC	CAC	18s rRNA	UBQ	CYP	18s rRNA
	0.91	0.76	0.85	0.65	1.15	0.69	1.23

**Note:** The expression stability values of the 12 reference genes and their rankings, as calculated by NormFinder, in total (A), different tissues (B), under abscisic acid treatment (C), under PEG treatment (D), under NaCl treatment (E), under heat treatment (F), and under cold treatment (G). A lower average M value indicates more stable expression.

### BestKeeper analysis

BestKeeper is another Microsoft Excel-based tool for stability analysis of candidate reference genes. With BestKeeper, the original average C_q_ values are used instead of transformed values. This algorithm compares the gene expression stability of all candidate reference genes overall. According to the BestKeeper analysis, *ACT* and *GAPDH* were highly stable, whereas *CAC* and *SAND* were the least stable under abscisic acid treatment. *Helicase* and *EF1-α* were the most stable, while *SAMDC* and *CAC* were the least stable in PEG-treated tissues. In NaCl-treated samples, the most stable genes were *GAPDH* and *helicase*, and the least stable were *PP2A* and *SAND*. *GAPDH* and *helicase* had high expression stabilities while *CYP* and *TUB* had the lowest expression stability under heat treatment. *GAPDH* and *βTUB* were the most stable, while *CAC* and *SAND* genes were the least stable under cold stress. *ACT* and *GAPDH* were highly stable in all tissues under all conditions (Table 4).

**Table 4:** Ranking of 10 reference genes in order of their expression stability, as calculated by BestKeeper.

Gene	Total(A)	Tissue(B)	ABA(C)	PEG(D)	NaCl(E)	Heat(F)	Cold(G)
*ACT*	0.988	0.974	0.998	0.933	0.990	0.986	0.992
*CAC*	0.866	0.937	0.242	0.719	0.886	0.881	0.973
*CYP*	0.901	0.871	0.888	0.952	0.966	0.232	0.998
*EF1a*	0.983	0.964	0.971	0.981	0.990	0.911	0.988
*GAPDH*	0.987	0.945	0.997	0.890	0.992	1	1.00
*helicase*	0.983	0.895	0.980	0.974	0.998	0.998	0.998
*PP2A*	0.948	0.961	0.978	0.945	0.805	0.985	0.999
*SAMDC*	0.951	0.880	0.975	0.791	0.970	0.983	0.985
*SAND*	0.855	0.823	0.229	0.667	0.848	0.957	0.935
*TUB*	0.952	0.878	0.961	0.972	0.923	0.804	0.999

**Note:** The expression stability values of 10 reference genes and their ranking, as calculated by BestKeeper, in total (A), in different tissues (B), under abscisic acid treatment (C), under PEG treatment (D), under NaCl treatment (E), under heat treatment (F), and under cold treatment (G).

### Reference genes validation

**Fig 4:**
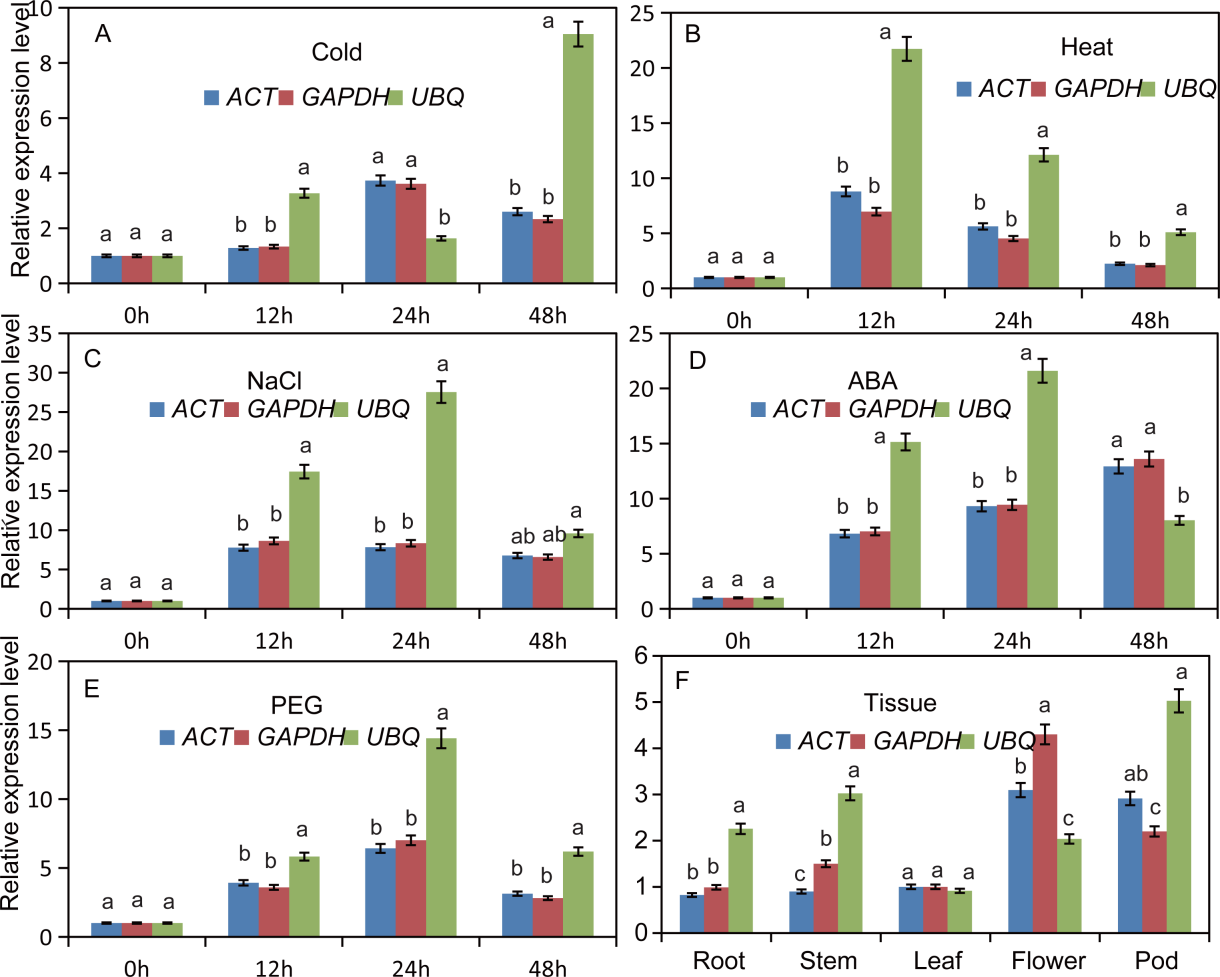
Validation of *NAC2* expression was assessed using individual reference genes (*ACT*, *GAPDH* or *UBQ*) under diverse abiotic conditions, (A) 4°C for 0, 12, 24 and 48h, (B) 40°C for 0, 12, 24 and 48h, (C) NaCl (200 mM), (D) ABA (150 mM), (E) PEG (100%), (F) Tissues (Root, Stem, Leaf, Flower and Pod).

**Fig 5:**
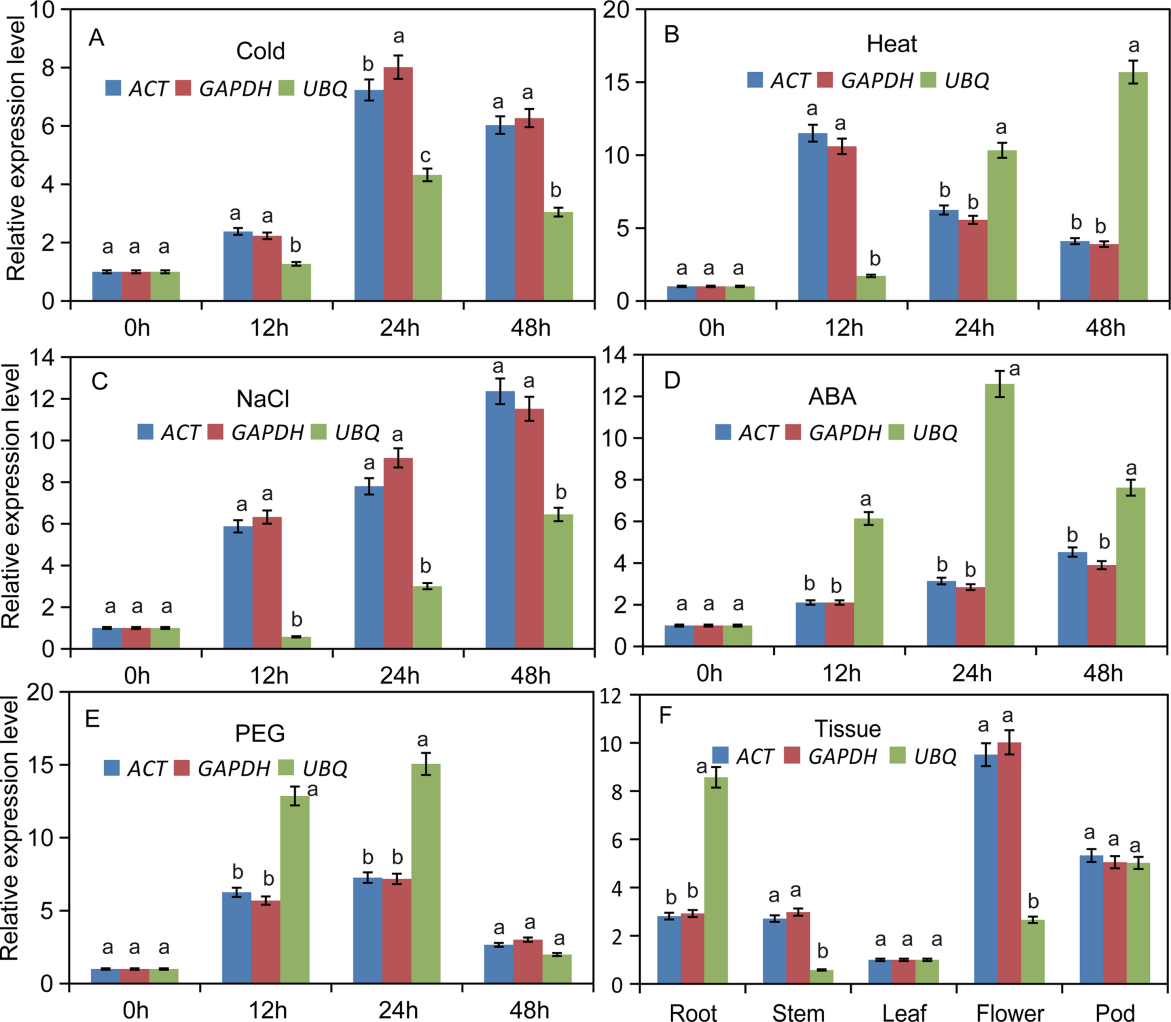
Validation of *BGL2* expression was assessed using individual reference genes (*ACT*, *GAPDH* or *UBQ*) under diverse abiotic conditions, (A) 4°C for 0, 12, 24 and 48h, (B) 40°C for 0, 12, 24 and 48h, (C) NaCl (200 mM), (D) ABA (150 mM), (E) PEG (100%), (F) Tissues (Root, Stem, Leaf, Flower and Pod).

To confirm expression stability of significantly normalized reference genes, it is crucial to calculate the relative expression levels of selected normalized genes. For this purpose, the expression level of the top two reference genes identified in this study, *ACT* and *GAPDH*, were compared along with the expression level of *NAC*2 (4A-F) and *BGL2* (5A-F). Highest expression of *NAC*2 was observed on ABA treatment at 48 h (Fig 4B), while decreased expression levels were observed in response to cold treatment at 12 h (Fig 4A). In the case of *BGL2*, highest expression was observed following NaCl treatment at 48 h (Fig 5C), while decreased expression level was observed following PEG treatment at 48 h (Fig 5E).

**Fig 6:**
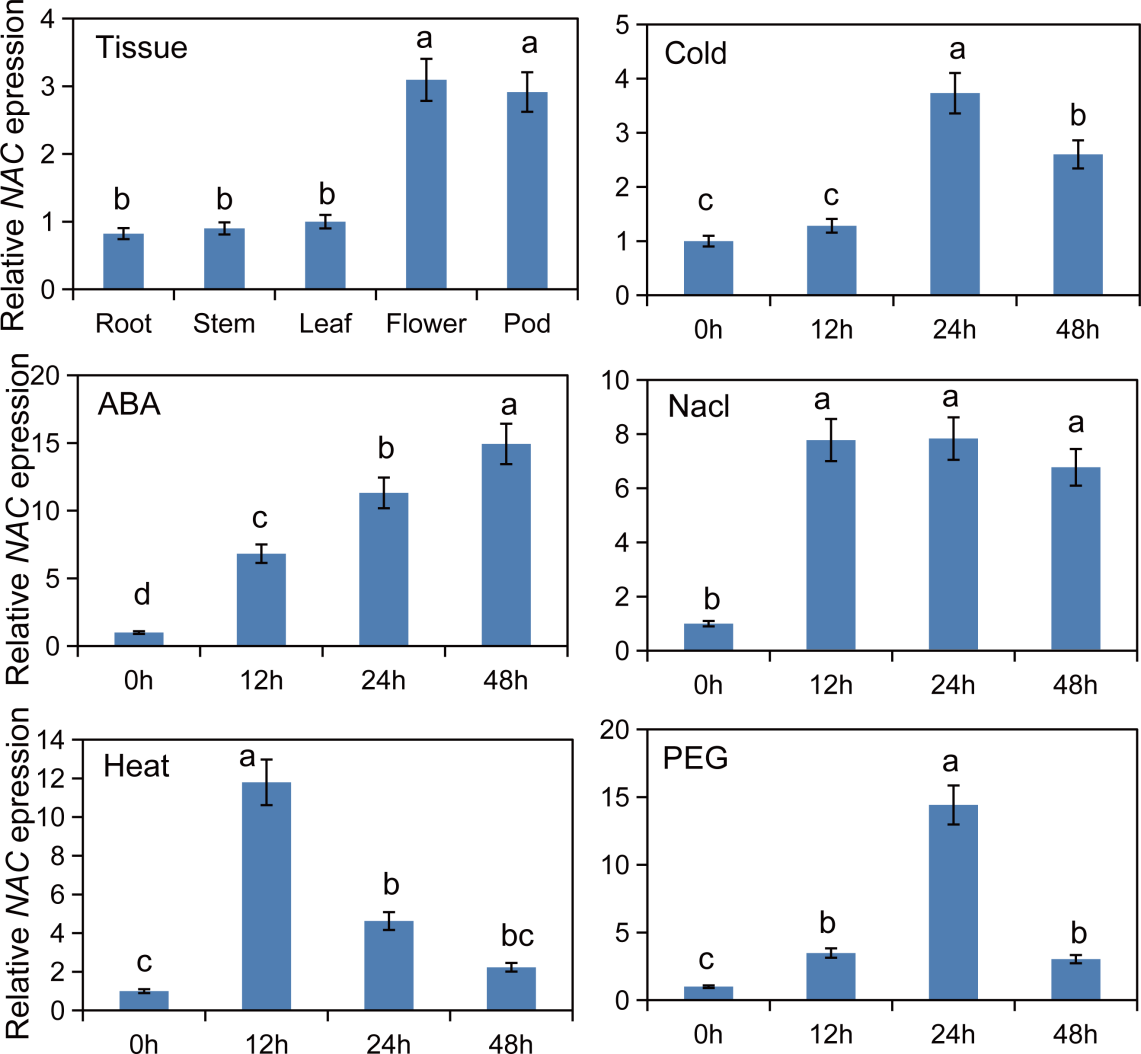
Expression profile of *NAC2* gene in different tissues and in response to different stressors in *Robinia pseudoacacia* as investigated by qPCR with *ACT* and *GAPDH* used as dual reference genes.

There were no significant differences in the expression patterns of *NAC2* and *BGL2* using either *ACT* or *GAPDH* as the internal control (P<0.05), which shows that *ACT* and *GAPDH* were suitable reference genes. In contrast, use of the least stable candidate reference gene (*UBQ*) with both target genes resulted in expression profiles that were unstable in all stress conditions and tissues (Figs 4 and 5). Expression profile of *NAC2* gene was significant when evaluated at P<0.05 (Fig 6).

## Discussion

Drought, salinity, heat, cold and other abiotic stress conditions have significant effect on black locust and other forest fauna. To improve resistance against these stress conditions and enhance plant productivity, it’s imperative to explore genes responsive to these stimuli. RNA sequencing and qPCR have been considered reliable techniques for the analysis of gene expression patterns. To obtain accurate results by qPCR, the PCR conditions and selection of genes used as internal controls must be optimized. The selection of an appropriate reference gene is a crucial prerequisite for gene expression analysis [46]. Internal control genes must retain expression stability, while the gene of interest exhibits varied in expression under the experimental condition.

Although *UBQ* showed highly stable expression in the model plant Arabidopsis [29,30], its expression varied across different developmental stages of rice and soybean [47,48]. The ribosomal RNA subunits 18S and 28S are commonly used internal control genes. While both genes are considered reference genes with extremely stable gene expression in all tissues of all organisms, their stability is controversial. We used mRNA for the RNA quantification studies, as mRNA free from other RNA residues gives satisfactory results. On the other hand, entire RNA contains variable amounts of 5S, 18S and 28S ribosomal RNA (rRNA) that may cause significant variations in results [38]. The most serious drawback of using these genes is their abundance compared with target transcripts. Because rRNA is not polyadenylated, these genes are not applicable when the template consists of cDNA reverse-transcribed from total RNA using oligo-dT primers. It is difficult to subtract the rRNA expression value from that of the gene of interest for accurate analysis of qPCR data. In short, for these reasons, 18S and 28S rRNA genes fail to replace other reference genes. There are no universal endogenous control genes available for gene expression profiling among all organisms. Thus, it is imperative to identify suitable reference genes as internal controls for accurate quantification of gene expression.

In this study, we selected and evaluated 12 genes from different functional classes and gene families as candidate reference genes i.e., *SAMDC*, *18s rRNA*, *EF1α*, *UBQ*, *βTUB*, *CYP*, *SAND*, *CAC*, *PP2A* and *helicase* for black locust gene expression studies among different tissues and abiotic stress treatments. Because these genes already have been used for gene expression profiling of other organisms, we also considered these genes as candidate references. For example, *ACT* has been used for qPCR studies in *Platycladus orientalis* [43], *TUB* in *Salix matsudana* [49], *UBQ* in longan tree [24], and *EF1β* and *GAPDH* in *Vernicia fordii* [50]. We selected these genes from RNA sequencing data (PRJNA260115) and used qPCR together with statistical and graphical techniques (geNorm, BestKeeper and NormFinder) to determine the most stable internal control gene for qPCR studies.

On the basis of three algorithms analysis results, we found *ACT* and *GAPDH* with most stable gene expression among whole plants, as well as different tissues under both stress and normal conditions (Table 5). Actin (*ACT*) is a cytoskeleton structural protein involved in cell signaling while glyceradehde-3-phosphate dehydrogenase (*GAPDH*) plays pivotal role in energy yielding step during carbohydrates metabolism and involved in DNA repair [51,52]. These results suggest that our discovery is perfectly in agreement with previous studies, such as *ACT* has been ranked as best internal control for gene expression studies in *Platycladus orientalis*, *Vernicia fordii* and *Pinus massoniana* [43,50,53], whereas it’s expression stability is unstable in *Glycine max* [10]. Similarly, *GAPDH* retained perfectly stable expression in *Vernicia fordii* and *Camellia sinensis* [50,54] in different tissues and under various abiotic stress conditions, whereas it’s expression was compromised in *Platycladus orientalis* [43]. In order to meager errors, it is highly recommended to use more than one appropriate internal control genes in qPCR studies [38]. In this study we wittily identified two most stable internal control genes such as *helicase* and *EF1α* under PEG stress, *GAPDH* and *βTUB* under cold, *GAPDH* and *helicase* under NaCl and heat while *ACT* and *GAPDH* under all conditions except heat.

**Table 5:** Ranking of the candidate reference genes according to their stability values using geNorm, NormFinder, and BestKeeper.

Gene Name	geNorm		NormFinder		BestKeeper*	
	Stability Value	Ranking Order	Stability Value	Ranking Order	Stability Value	Ranking Order
*ACT*	0.011	1	0.016	1	0.988	1
*GADPH*	0.013	2	0.017	2	0.987	5
*PP2A*	0.019	3	0.037	5	0.948	7
*TUB*	0.025	4	0.059	7	0.952	10
*EF1α*	0.030	5	0.030	4	0.983	4
*CYP*	0.034	6	0.061	8	0.901	3
*Helicase*	0.039	7	0.017	3	0.983	6
*SAMDC*	0.044	8	0.047	6	0.951	8
*18SrRNA*	0.056	9	0.091	12	—-	-
*UBQ*	0.065	10	0.077	11	—-	-
*SAND*	0.076	11	0.073	10	0.855	9
*CAC*	0.084	12	0.070	9	0.866	2

Note

*In the BestKeeper analysis, the *18s rRNA* and *UBQ* genes were rank-ordered according to their CV [% C_q_] and SD [6 **C**_**q**_].

Considering the importance we selected two genes, *NAC2* and *BGL2* as target genes for validation because both of these target genes are involved in flowering and abiotic stress responses. Target genes expression was very accurately measured when two selected reference genes *ACT* and *GAPDH* were tested along with targets. But, the expression of target genes was extremely unstable when its expression was calculated as compared with the least stable candidate reference gene *UBQ* (Figs 4 and 5). Our findings deliberately support previous discoveries that in order to normalization usage of two highly stable internal reference genes has significant importance. Moreover, two internal control gens are perfectly enough to analyze gene expression as has been done earlier in case of *Salix matsudana* [49] and *Platycladus orientalis* [43]. Therefore, *ACT* and *GAPDH* are the most stably expressed reference genes and are highly recommended in single or both as endogenous controls for gene expression studies under both normal and stress conditions. This study has important implications in obtaining more accurate results from expression analyses of black locust.

In conclusion, out of the 12 black locust genes evaluated, the two most reliable internal reference genes were *ACT* and *GAPDH*. All other candidate genes were not deemed suitable internal controls for black locust, because they exhibit high expression diversity. To obtain the most reliable results in gene expression studies of black locust, *ACT* and *GAPDH* are recommended as internal controls for qPCR analysis.

## Supporting information

S1 Fig Relative expression levels of the *NAC2* in plants treated with Cold, ABA, NaCl, Heat, and PEG stresses. (TIF)

S2 Fig Relative expression levels of the *NAC2* in different tissues. (TIF)

S3 Fig Relative expression levels of the *BGL2* in plants treated with cold, ABA, NaCl, Heat, and PEG stresses. (TIF)

S4 Fig Relative expression levels of the *BGL2* in different tissues. (TIF)

S1 File Selection of *NAC2* for validation of results. (DOCX)

S2 File Selection of *BGL2* for the validation of results. (DOCX)

S3 File Validation of results by using *NAC2* gene. (DOCX)

S4 File Validation of results by using *BGL2* gene. (DOCX)
