# Supplementary figures and images for: Selection and validation of reference genes for quantitative gene expression analyses in black locust (Robinia pseudoacacia L.) using real-time quantitative PCR
Source: PLoS One. 2018 Mar 12;13(3):e0193076. doi: 10.1371/journal.pone.0193076 (PMC5846725; doi:10.1371/journal.pone.0193076)

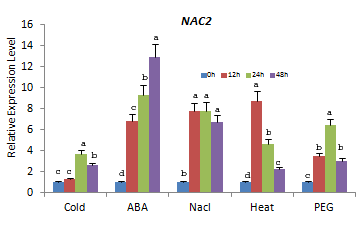

Supplement: S1 Fig — (TIF) [file pone.0193076.s001.tif]

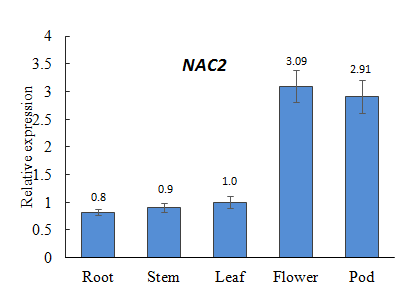

Supplement: S2 Fig — (TIF) [file pone.0193076.s002.tif]

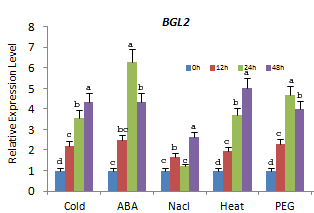

Supplement: S3 Fig — (TIF) [file pone.0193076.s003.tif]

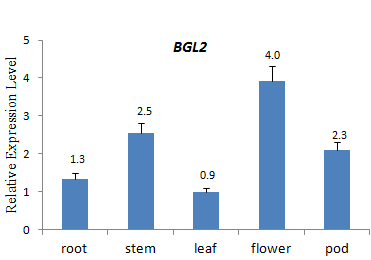

Supplement: S4 Fig — (TIF) [file pone.0193076.s004.tif]
