# Supplementary material for: Selection and validation of reference genes for quantitative gene expression analyses in black locust (Robinia pseudoacacia L.) using real-time quantitative PCR
Source: PLoS One. 2018 Mar 12;13(3):e0193076. doi: 10.1371/journal.pone.0193076 (PMC5846725; doi:10.1371/journal.pone.0193076)
Supplement: S1 File — (DOCX) [file pone.0193076.s005.docx]

**S1 File. Selection of *NAC2* for validation of results**

Out of all 58, the largest plants transcription factors protein family is *NAC* transcription factors gene family (http://planttfdb.cbi.pku.edu.cn/). It is involved in senescence of flowers and leaves [1,2], number of tillers [3], development of root and shoots apical meristem [4-8] as well as lateral roots development [9,10]. *NAC* transcription factors gene family also plays key role in xylogenesis, fiber, embryo and wood development [11-12], hormone signaling [9,13-15], flowering induction under stress response [16,17], cell cycle and determining cell fate [18,19], remobilization of grain nutrients [20], determination of shoot branches [21], biotic and abiotic stress [22-32] in plants.
