# Supplementary material for: Selection and validation of reference genes for quantitative gene expression analyses in black locust (Robinia pseudoacacia L.) using real-time quantitative PCR
Source: PLoS One. 2018 Mar 12;13(3):e0193076. doi: 10.1371/journal.pone.0193076 (PMC5846725; doi:10.1371/journal.pone.0193076)
Supplement: S2 File — (DOCX) [file pone.0193076.s006.docx]

**S2 File. Selection of *BGL2* for the validation of results**

*BGL2* (*beta-1,3-glucanase 2*) is involved in flowering [33] and it is the homologue of *Arabidopsis thaliana* (AT3G57260). We used already batch uploaded transcriptome data of our lab in NCBI database (PRJNA260115) for the primer designing (Table 1).
