# Supplementary material for: Selection and validation of reference genes for quantitative gene expression analyses in black locust (Robinia pseudoacacia L.) using real-time quantitative PCR
Source: PLoS One. 2018 Mar 12;13(3):e0193076. doi: 10.1371/journal.pone.0193076 (PMC5846725; doi:10.1371/journal.pone.0193076)
Supplement: S3 File — (DOCX) [file pone.0193076.s007.docx]

**S3 File. Validation of results by using *NAC2* gene**

To confirm expression stability of significantly normalized reference genes, it is crucial to calculate the relative expression levels of selected normalized genes. For this purpose, the expression level of top two reference genes identified in this study, *ACT* and *GAPDH*, were compared along with the expression level of *NAC* transcription factor *AtNAC2* (At5g36910). Highest expression of *NAC2* gene was observed on ABA treatment at 48 h, while decreased expression level was observed on cold treatment at 12 h (Fig 1). While, we analyzed the expression pattern of *NAC2* transcription factor in different tissues of black locust, we found the highest expression level in flowers (Fig 2). It is the first evidence of results accuracy.
