# Supplementary material for: Selection and validation of reference genes for quantitative gene expression analyses in black locust (Robinia pseudoacacia L.) using real-time quantitative PCR
Source: PLoS One. 2018 Mar 12;13(3):e0193076. doi: 10.1371/journal.pone.0193076 (PMC5846725; doi:10.1371/journal.pone.0193076)
Supplement: S4 File — (DOCX) [file pone.0193076.s008.docx]

**S4 File. Validation of results by using *BGL2* gene**

*BGL2* gene was used as the second gene for validation of results. There were no significant differences in the expression patterns of *NAC2* and *BGL2* using either *ACT* or *GAPDH* as the internal control (P>0.05), which shows that *ACT* and *GAPDH* were suitable as reference genes. While, the highest gene expression level of *BGL2* gene was also observed on ABA treatment at 24 h and decreased expression was observed on NaCl treatment at 12 h (Fig 3). While compared the *BGL2* gene expression in different tissues we observed highest gene expression in flowers which is another evidence of peculiar nature of our results (Fig 4). Hence, it’s proved that *ACT* and *GADPH* are best suited pair of genes for internal control in qPCR studies of black locust.
